# The life and work of Isidor Fischer (1868–1943)—A pioneer of the social history of medicine

**DOI:** 10.1007/s00508-025-02616-5

**Published:** 2025-09-19

**Authors:** Josef Hlade

**Affiliations:** Institut für Ethik, Sammlungen und Geschichte der Medizin, Medizinische Universität Wien, Währinger Straße 25, 1090 Wien, Austria

**Keywords:** Vienna Medical School, Institutionalization of medical history, Anti-Semitism, Ignaz Semmelweis, History of gynecology and obstetrics

## Abstract

Isidor Fischer was one of the most important medical historians of the interwar period and one of the most important Austrian medical historians in general. He wrote several classics of medical history that remain fundamental today. His *Biographical Encyclopedia of Outstanding Doctors of the Last Fifty Years *(1932–1933), for example, was a major achievement in medical history and, until today, no medical history library could do without it; however, his influence on the social history of medicine, a field that only began to evolve in the 1920s, has not yet been adequately recognized. This is due, among other reasons, to the fact that Fischer, who was of Jewish descent, experienced anti-Semitism throughout his career. His *History of the College of Physicians* was published before the Nazi takeover of Austria, but without mentioning his name. A few months later, following the *Anschluss *(annexation) he was forced to flee Austria. His life is paradigmatic of Austrian medical history in the interwar period. His Jewish descent, coupled with repeated accusations that he pursued an academic career in medical history to gain financial advantage for his gynecological practice, resulted in his being denied an associate professorship. An extensive study of archival materials, mainly in Viennese archives, now makes it possible for the first time to draw a comprehensive picture of Fischer’s work and to explain why, despite his considerable achievements as a medical historian, Fischer was denied a career and described as an outsider of medical history in Vienna.

## Introduction

**Fig. 1:**
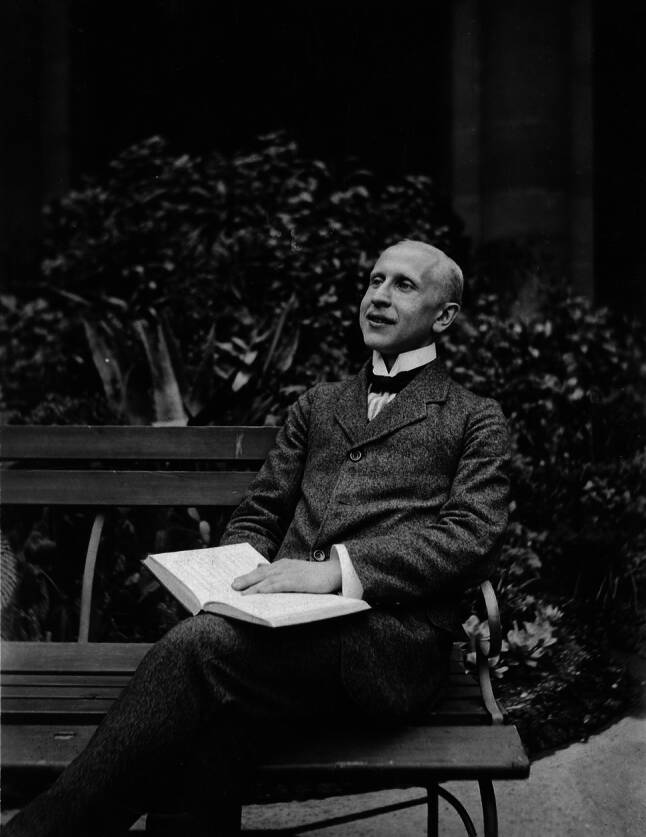
Isidor Fischer (1868–1943). Source archives of the University of Vienna (Archiv der Universität Wien, hereafter: UAW), photograph collection (*Fotosammlung*) 106.I.4071

Isidor Fischer was an internationally renowned medical historian. The British historian of medicine, Charles Joseph Singer (1876–1960), called him “one of the greatest living authorities on the history of medicine.”^1^ Fischer’s theoretical approach was similar to that of Max Neuburger (1868–1955), the founder of the Vienna Institute for the History of Medicine and holder of the Chair of the History of Medicine in Vienna from 1917 onward. Neuburger and Fischer’s work was central to the establishment of modern medical historiography, as they acted as pioneers of a history of medicine conceived of as a theory of science, an approach that was later adopted especially in English-speaking countries [1–4]; however, despite the fact that Fischer is considered one of the most important medical historians of the interwar period [5, 6], his work has not yet been sufficiently recognized. Voswinckel, who has undertaken a major project to continue Fischer’s *Biographisches Lexikon der hervorragenden Ärzte der letzten fünfzig Jahre* (Biographical Encyclopedia of Outstanding Doctors of the Last Fifty Years), has provided us with a great deal of important information on Fischer’s biography [7]. In addition, Weindling conducted important research into Fischer’s time spent in exile in the UK [2]. Zeitlhofer first and extensively researched Fischer’s work as a librarian at the College of Physicians in Vienna (Gesellschaft der Ärzte in Wien) and provided an overview of his contributions to medical history [8]; however, an extensive study of archival materials, mainly conducted in Viennese archives, now makes it possible for the first time to draw a comprehensive picture of Fischer’s work and to explain why, despite his considerable achievements as a medical historian, Fischer was denied a career and described as an outsider of medical history in Vienna. The medical historian Marlene Jantsch (1917–1994), who may have recognized similarities between his fate and her own, called him “the stepchild (Stiefkind) of Viennese medical history”^2^ (Fig. 1).

## A gynecologist and medical historian

Fischer was a gynecologist by profession but he also took a particular interest in the history of his discipline early on and gradually began to focus his work on the history of medicine; however, as he never managed to obtain a funded position in the field of medical history, he was always dependent on his day job as a gynecologist.^3^

Fischer was born in Vienna on 29 September 1868 and studied medicine at the University of Vienna. Following his graduation in early 1892, Fischer worked at the Vienna General Hospital in the clinics of Hermann Nothnagel (1841–1905), Moritz Kaposi (1837–1902), and Josef Weinlechner (1829–1906). He also underwent specialist training in gynecology with Rudolf Chrobak (1843–1910) in Vienna and August Martin (1847–1933) in Berlin. From 1897 to 1921, he served as director of the outpatient clinic “Frauenkrankeninstitut Charité” in Vienna.^4^ He resigned as director in 1921 in order to devote as much time as possible to the history of medicine.^5^

In a curriculum vitae dated 1939, Fischer listed publications totalling 185 papers and 11 books.^6^ Fischer’s initial focus was on the history of his own discipline, gynecology. His first major publication, *Geschichte der Geburtshilfe in Wien *(History of Obstetrics in Vienna), appeared in 1909. It remains the most important work on the history of gynecology during this period of Viennese medicine and is still used as a reference work [9, 10]. Fischer’s work on the history of medicine attracted the interest of Max Neuburger, then associate professor and later full professor, who initiated Fischer’s habilitation with this publication. After his habilitation in 1914, Fischer increasingly focused his work on the history of medicine.

An important role is played in Fischer’s “History of Obstetrics in Vienna” by Ignaz Semmelweis (1818–1865). Fischer carried out extensive research into Semmelweis in the university deanery files, and his biographical account is probably the most significant from this period alongside that by Fritz Schürer von Waldheim (1866–1935) from 1905 [11]. Fischer endeavored to contextualize Semmelweis’s discovery of the antiseptic principle and described it against the backdrop of the controversies of the time [12]. He also explored the reasons why Semmelweis’s findings were unable to gain acceptance at a time when bacteriology was not yet known, and the detection of the infectious agent was not possible:

“The new doctrine was not yet sufficiently prepared. The empirically obtained data did not yet speak eloquently enough, and Semmelweis had not yet been able to elucidate the deeper connections that we can clearly see today after a long period of painstaking work” [13].

Erna Lesky’s thesis that Semmelweis’s discovery was a fruit of the Second Vienna Medical School [14] had also already been laid out by Fischer. Fischer had already referred to Semmelweis’s studies with the pathologist Karl Rokitansky (1804–1888) and the dermatologist Ferdinand Hebra (1816–1880) and to the fact that in 1847 he applied for an assistant position with Josef Škoda (1805–1881), who had just become professor of internal medicine. Semmelweis’s extensive pathological-anatomical studies under Rokitansky, while also emphasizing the significant support Semmelweis received from the most important representatives of the “Second Vienna Medical School”:

“If we now once again recall those men who, like Hebra and Skoda, were imbued with the correctness of Semmelweis’s views from the very first moment, we cannot say, as is so often claimed, that Semmelweis found no understanding and no support in Vienna” [13].

## Fischer’s contribution to the institutionalization of medical history in Vienna and to a sociomedical approach

The Chair of Medical History in Vienna is one of the oldest in Europe and was once held by prominent medical historians such as Franz Romeo Seligmann (1808–1892) and Theodor Puschmann (1844–1899); however, it took many years after Puschmann’s death before the chair was filled again and his long-held wish to create an Institute for the History of Medicine was realized. Puschmann’s most important students were Neuburger and Robert von Töply (1856–1947), who was a military doctor by profession. Both received their habilitation in 1897/1898 and were appointed as associate professors in 1904. On 5 July 1914, Neuburger was finally recommended ad personam by the professorial council as full professor of medical history, although the appointment was delayed until late 1917.^7^ Neuburger’s appointment was a significant step in the institutionalization of the subject in Vienna but the location of the Institute for the History of Medicine, which would also function as a museum with a large collection of medical artefacts, had not yet been decided [15, 16].

In 1920, Fischer was instrumental in the founding of the Vienna Institute for the History of Medicine. By this time, Fischer was the most important medical historian in Vienna alongside Neuburger, as Töply had retired from the field in 1912.^8^ Fischer’s reputation as a historian of medicine and his good contacts with influential professors such as the surgeon Anton von Eiselsberg (1860–1939), President of the College of Physicians, enabled him to contribute to the founding of the institute. Fischer later pointed out to Neuburger that he had “taken the most active intellectual interest in the creation of the institute […] from the very beginning.”^9^

Eiselsberg had already intervened on Neuburger’s behalf at the Ministry of Education in 1917 in the appointment procedure for the professorship and had thus greatly facilitated the appointment.^10^ On 20 July 1920, a meeting took place at the College of Physicians that turned out to be decisive for the foundation of the history of medicine in Vienna. The three authorities on the history of medicine spoke during the discussion at this meeting: Neuburger, Adolf Kronfeld (1861–1938), the editor-in-chief of the *Wiener Medizinische Wochenschrift*, who published works on the history of medicine in addition to his main activities, and finally Fischer. The initiative was crowned with success: In late August 1920, the transfer of the collection from the First Medical Clinic, where the institute and the collection were provisionally housed, to the Josephinum finally began [17].

On this memorable occasion, Fischer felt compelled to make a general plea for the importance of medical history to the field of medicine, noting a “lack of historical awareness” among physicians, which he thought led to an “absence of a distinctive sense of belonging to the medical profession.” This observation is significant in light of the location of his speech, which was delivered in the ceremonial hall of the College of Physicians, a central institution in the medical community during this era. Notably, Fischer had dedicated a significant portion of his research activities not only to the College of Physicians but also to the historical development of the medical profession in the latter half of his career [8, 31].

Fischer’s above-cited statement reveals his distinctive theoretical approach, characterized by a “genetic evolutionary method” based on the Viennese tradition. This approach was described by Neuburger as a “critical, philosophical view of history” [18] and was succinctly summarized by Max Seiffert (1865–1916) as an attempt “to visualize the development of research methods and the emergence of our current knowledge from the errors and mistakes […] of medical and scientific research in a genetic context” [19].

In his statement, Fischer emphasized:

“It is true that in medicine it is important to look forward, to penetrate deeper and deeper into the mysteries of disease, but it is nevertheless necessary for anyone who continues to build to look back from time to time at the foundation onto which he is consciously or unconsciously piling new blocks” [20].

Fischer rarely commented so explicitly on his methodological approach as a medical historian; however, on the occasion of the planned founding of the first Medical History Association in Vienna in 1932, Fischer once again reflected on this topic. He criticized “a jumble of isolated research results” in the history of medicine, which he contrasted with a history of medicine “striving for synthesis” as an ideal. The type of medical historiography he had in mind strove “to become clear about the origin and phenomenon of life, the mind-body problem, the nature of health and illness, the foundations of medical knowledge, and the limits of the medical art of healing.” In doing so, it was necessary “not to get lost in detailed research, but to outline the great intellectual development that medicine can look back on”. In addition to this method, which in his opinion had essentially been implemented by Neuburger, Fischer also “tried to grasp the ethical and social foundations of our professional practice in its historical development—taking into account the importance that professional medical issues and medical code of conduct have gained in our time” [21]. As the following overview of his publishing activities shows, Fischer pursued a sociomedical approach early on, which moved away from a focus on personal history and somewhat later became the focus of general interest through the research of Henry Ernest Sigerist (1891–1957) [2, 22].

## Fischer and the history of social medicine

After completing his first major work, his “History of Obstetrics in Vienna”, Fischer turned increasingly to cultural studies from 1909 onwards. The history of the medical profession and the history of social and occupational medicine were a major focus of his research, which remains important to this day [23, 24]. His book *Ärztliche Standespflichten und Standesfragen* (Medical Professional Duties and Ethics, Vienna/Leipzig 1912) [25] was published as early as 1912. Over the following years he also published a large number of articles on the subject [26, 27]. A central publication was the series of articles “Geschichte des Krankenkassenwesens” (History of the Health Insurance System), which ran to over 40 pages [28].

In 1913, Fischer launched the series “Beiträge zur Kulturgeschichte” (Contributions to Cultural History) in the *Wiener Klinische Wochenschrift*, in which the work of physicians of significance to cultural history was described at irregular intervals [29]. One of the first articles was dedicated to the physician and philosopher Johann Benjamin Erhard (1766–1827), whose major work “On the Right of the People to a Revolution”, published in 1795, was banned in several German states. Fischer showed how Erhard, under the influence of his Enlightenment positions, placed public healthcare, in the sense of what Foucault would later call “biopolitics,” at the center of his considerations and thus adopted an approach that first took systematic form with Johann Peter Frank’s (1745–1821) “A Complete System of Medical Policy” [30].

Fischer was also the first to publish important findings on the history of Viennese medicine [8, 31]. He provided the first evidence of the central role played by the medical profession at the beginning of the 1848 revolution in Vienna [8, 31–33]. Another article from this period was dedicated to the founding of the Austrian emergency medical services [34]. It was during this period that Fischer turned away from individual-centered historiography and towards a thematically oriented social history of medicine. In 1913 and 1914, the articles “Zur Geschichte der ärztlichen Schweigepflicht” (On the History of Medical Confidentiality) [35] and, on the occasion of the outbreak of war in 1914, “Der Krieg und die Wiener Ärzteschaft” (Viennese Doctors and the War) were published, in which the care of wounded and sick soldiers was interpreted as an expression of the importance of “caring for the welfare and well-being of the individual in the state” [8, 36].

Fischer also devoted himself to the social medical work of Leopold Schrötter von Kristelli (1837–1908), who was responsible for founding the first tuberculosis sanatorium in the Habsburg Monarchy and an association for schoolchildren suffering from tuberculosis [37]. In his *Geschichte der Gesellschaft der Ärzte in Wien *(History of the College of Physicians in Vienna), Fischer emphasized the society’s welfare achievements. For example, the construction of the Vienna Mountain Spring Pipeline in 1873, which supplied Vienna with fresh drinking water and thus significantly improved the hygienic situation of the poorest portions of the population in particular, was expressly acknowledged. Another topic was the discussion about the construction of tuberculosis sanatoriums for the destitute urban population. In 1913, when the convalescent home for orphans suffering from tuberculosis planned by the philanthropist Karl Kupelwieser (1841–1925) at the Semmering Pass was prevented by local community representatives and hoteliers, who feared that it would damage the area’s touristic image, the College of Physicians sent a memorandum to the Ministry of Education, which is quoted at length in Fischer’s book. This memorandum warned that the authorities’ refusal to approve Kupelwieser’s sanatorium was proving the country to be “extremely backward in the eyes of the entire world.” The sanatorium was therefore deemed necessary to prevent Austria “from disgracing itself in front of the entire educated world” [38, 39].

In 1939, the article “Geheimpolizeiberichte über Ärzte und Sanitätswesen in Wien aus den Jahren 1855–1857” (Secret Police Reports on Physicians and Medical Facilities in Vienna from 1855–1857) appeared in the journal *Janus* (43, 1939: 214–232), probably also inspired by the dangers of the Nazi regime.

In this article, Fischer turned his attention to political historiography. His focus lay on the activities of the secret police and their investigation of individuals deemed suspicious or dangerous to the state. Using police reports on representatives of the Second Vienna Medical School, Fischer illustrated the intense scrutiny they faced in the aftermath of the 1848 revolution, which resulted in extensive monitoring and surveillance. At the same time, Fischer drew attention to the anti-Semitism already widespread at the time, which was also evident in the secret police reports: “[The police reports] show us how little respect was shown to the medical profession at a time when the younger Viennese medical school was approaching its peak and performing at its best. They show us what people thought of the growing number of Jewish doctors […].” One of these reports, written by Police Commissioner Josef Nilius, stated the following: “Almost a third of physicians are either Jewish, descended from Jews, or baptized Jews. In the eyes of many, that alone is enough to discredit the entire profession.”

From the mid-1920s onward, Fischer also turned his attention to the history of Jewish medicine [40]. During the 1930s, he published several articles on this subject, providing insights into both the role of Jewish physicians in society and growing anti-Semitism. Fischer described the emergence of anti-Semitism in Anton Edler von Rosas’s (1791–1855) “Ueber die Quellen des heutigen aerztlichen Missbehagens” (On the Sources of Discontent in Medicine Today, Medicinische Jahrbücher 40, 1842), as well as the ensuing debate. During this debate, the Jewish writer Ignaz Jeitteles (1783–1843) and Rabbi Isaak Noah Mannheimer (1793–1865) argued against Rosas’s anti-Semitic views, supported by representatives of the Jewish community in Vienna [41, 42]. Fischer mainly wrote these articles for Jewish humanitarian organizations.

Finally, Fischer made a name for himself as the author of two encyclopedias, the importance of which is demonstrated by the fact that they are still being reissued and continued today. Fischer’s *Biographical Encyclopedia of Outstanding Doctors of the Last Fifty Years* (1932–1933) was a “top achievement in contemporary medical history” and remains an indispensable part of any medical library to this day [43]. It went through several editions (the most recent of which was published in 1962). Fischer himself had planned updates to the encyclopedia but was no longer able to carry them out. Since 2002, Peter Voswinckel has been expanding the encyclopedia completed in 1933 against the background of the documentation of the expulsion and murder by the National Socialists of a large number of the doctors featured in Fischer’s work. His aim is to augment the subsequent biographies of the doctors who were still alive when Fischer first completed the encyclopedia (comprising about half of the entries). The first part has already been published [44]. Voswinckel describes Fischer’s encyclopedia as “the most representative and comprehensive biographical reference work in the history of medicine to date […], without having found a comparable successor on an international scale” [44]. It remains a basis for research today both in its original form but also thanks to the additions made by Voswinckel [45–47]. In 1931, Fischer published the book *Der Eigennamen in Krankheitsterminologie* (Proper Names in Disease Terminology) [48], a dictionary of medical eponyms based on personal names. In the UK, where he spent his final years after fleeing persecution by the Nazis, he worked on a supplementary volume on geographical eponyms (*Geographical Names in Medical Terminology*), to which he devoted a large part of his time and which is still being continued today by British medical historians [49].

## Fischer’s failed attempt to obtain a professorship

It was evident that Neuburger prevented Fischer from pursuing a further career, which was in any case not very promising due to his Jewish descent and the difficult position of medical history within academia. Neuburger initially supported Fischer, who gained a habilitation in medical history in 1914 thanks to Neuburger’s intervention. Within just a few years, however, their relationship deteriorated considerably.^11^ At the height of the conflict between the two, Fischer accused Neuburger of “heavy-bloodedness, moodiness, and grumpiness.”^12^ Shortly after the successful founding of the Institute for the History of Medicine in 1920, the conflict finally escalated, with Fischer identifying a central dispute as the cause of the final rift. On 12 September 1922, Fischer wrote to Neuburger expressing his perspective on the matter:

“Just remember what happened a year and a half ago. The scene that I will never forget took place in the catalog room of the university library. I timidly asked if I could also give my lecture at the ‘Institute’. Do you remember the outburst of unjust reproaches and undeserved abuse that I received in response to this question, an outburst that not even a half-baked grammar school student gets to hear from his choleric teacher? I kept quiet, but having faced many adversities in my life, I had enough dignity and self-confidence left to avoid approaching you in future and exchanging ideas, although that would certainly have been to my advantage; indeed, it was made impossible for me to ever enter the ‘Institute’ again, in the creation of which I had taken the most active intellectual part from the very beginning.”^13^

Fischer was largely dependent on Neuburger’s support in his endeavors to obtain an associate professorship in the history of medicine and therefore asked him several times to recommend him to the College of Professors. On 20 April 1924, despite the ongoing conflict, he sent the following letter to Neuburger:

“As it is now exactly ten years since I was able to complete my habilitation at your suggestion and through your intervention, I would like to ask whether it would be convenient for you to suggest my appointment [as associate professor] to the College of Professors.

With the exception of the war years, I have taught regularly, given a number of lectures, and published a large number of smaller and larger publications.

With regard to my practice, I strictly adhered to the agreement that I should not use misleading designations on signs, prescriptions, in the telephone directory, etc. I resigned from my position at the ‘Charité’ and generally restricted my practice in favor of my scientific work to the extent necessary for the life of my family.

The professors of obstetrics and gynecology will also be able to testify that I have practiced in the most respectable manner—far from any advertising, far from abortion.

In Germany, two colleagues who habilitated after me, Schmitz (Bonn)^14^ and Haberling (Cologne)^15^, are already professors.

Do not take offense at these lines of mine—they are not the result of vanity—but consider them a request that you can safely throw in the wastepaper basket if you do not consider them justified.”^16^

By the mid-1920s, the pugnacious Neuburger had also fallen out with the leading German historian of medicine, Karl Sudhoff (1853–1938). While Fischer continued to attend conferences of the German Society for the History of Medicine and Science from the second half of the 1920s onward, Neuburger was virtually isolated during this period.^17^ Even before his habilitation in 1914, Fischer had maintained contact with Sudhoff and was a regular guest at the society’s meetings, and Sudhoff in turn took an interest in Fischer’s career.^18^ In late March 1914 Neuburger had informed Sudhoff that Fischer had successfully completed his probationary lecture.^19^ At the time, Neuburger, who had not yet been appointed full professor himself, clearly saw Fischer as part of a network that would put the history of medicine on a firm footing both in the German-speaking world and internationally. By mid-July, however, he was already making negative remarks to Sudhoff about Charles Singer (1876–1960) and Fischer:

“Dr. Charles Singer from Oxford was here a few days ago. He seems to be a capable person, but the high self-confidence of today’s young people is somewhat striking; Klebs [Arnold Carl Klebs (1870–1943)] is much more likeable to me. I don’t have good experiences with Fischer either, whom I have supported.”^20^

In 1929 there was a reconciliation between Sudhoff and Neuburger, and it was probably Sudhoff’s intervention with Neuburger that led to the latter finally deciding, on 29 November 1929, to recommend Fischer for a professorship in the College of Professors.^21^ As they had just reconciled, Neuburger did not want to jeopardize his friendship with Sudhoff again. Thus, it was this reconciliation that was decisive for Neuburger’s proposal:

“I would very much appreciate it if you could decide to lobby the Vienna faculty to award your colleague Isidor Fischer the title of professor. The whole world expects this of you and it would be appreciated as a great human touch.”^22^

On 5 December 1929, Neuburger wrote to Sigerist that he had recommend Fischer “for the award of the title of professor,” despite the “superhuman effort” this required of him.^23^ Nevertheless, Neuburger’s proposal was rejected by the responsible committee and on 5 February 1930, it was decided not to present the proposal at the meetings of the College of Professors.^24^

**Fig. 2:**
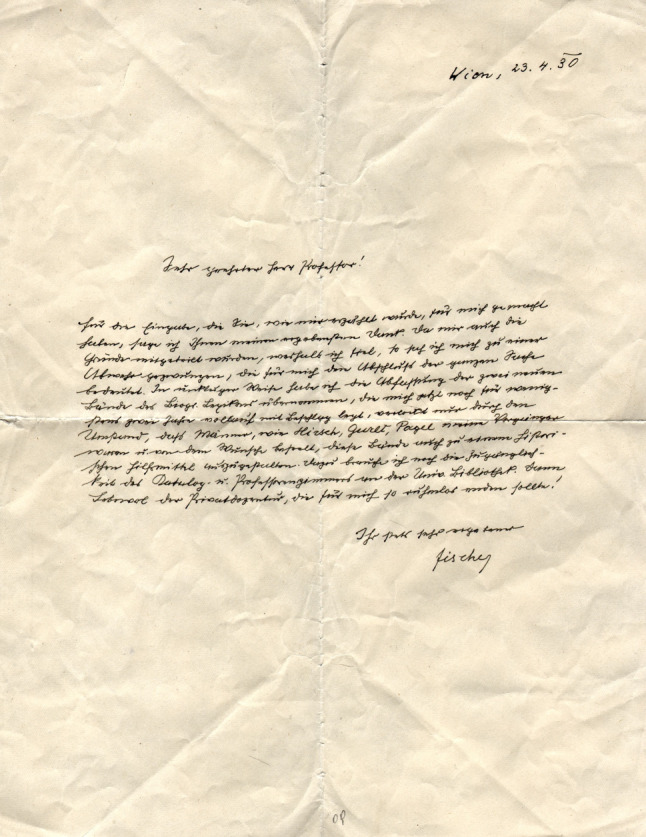
MUW-AS-006014-F-0035-009. Fischer to Neuburger, Vienna, 23 April 1930. In this letter, Fischer informed Neuburger of his plans to publish a “defense” against the reasons given by the College of Professors for not recommending him as a titular professor

Fischer was frequently subjected to massive hostility and had already been advised to resign from the management of his gynecological outpatient clinic during his habilitation. He was accused of trying to obtain the title of lecturer, which he was awarded in 1914, in order to use it as advertising for his medical practice.^25^ Fischer chose not to appeal but decided to publish a counterstatement. In a letter to Neuburger, he explained: “Since I was notified of the reasons why I failed, I felt compelled to make a defense, which for me meant the end of the matter”^26^. Neuburger was not too sad about Fischer’s rejection, but rejected any responsibility for it, and claimed that Fischer agreed with this view, as Neuburger was not a member of the “standing commission” that had rejected Fischer^27^ (Fig. 2).

The document published by Fischer shows that he was accused of having “chased after” a gynecological practice for monetary reasons. Furthermore, it was claimed that he had sought his private lectureship and the desired title of titular professor for “advertising purposes” and due to a “business acumen”^28^. Such accusations were typical strategies employed by anti-Semitic networks to hinder the careers of Jewish scientists [50–52]. Fischer himself tried to defend himself by pointing out that he was unable to earn a regular income as a medical historian and was therefore forced to run a private gynecological practice.^29^ In 1932, Fischer ultimately had his title of “private lecturer” removed from the directory of medical specialists in the telephone directory. The reason given for this was a complaint made in the College of Professors, which stated that Fischer had misused for “personal advertising” his contribution to the medical history chapter of a travel guide published by Alexander Mehan.^30^

**Fig. 3:**
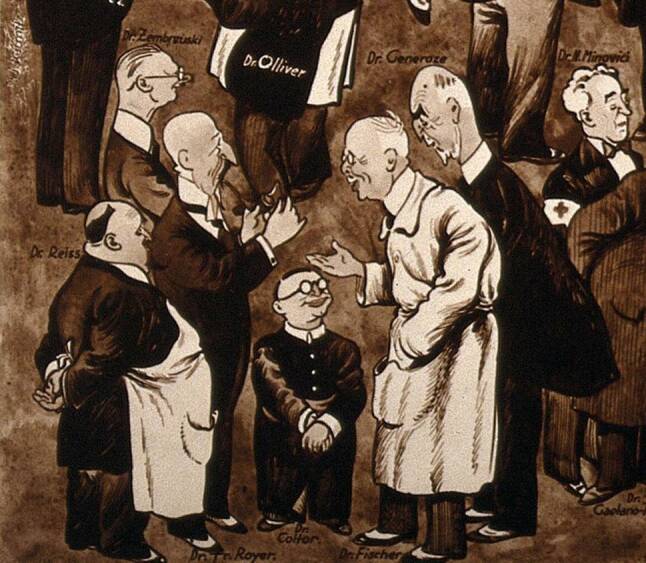
Caricature of the participants at the International Congress for the History of Medicine, Bucharest 1932 (“Delegates in Conversation”). Source: Wellcome Collection, 568634i. Fischer (in the foreground on the right, wearing a white coat) in conversation with other congress participants. Fischer was a frequent guest at medical history congresses and was the most important representative from Vienna at the time, while Neuburger was isolated from the community because of his dispute with the German medical historian Karl Sudhoff (1853–1938)

Fischer attributed the hostility from his colleagues to, among other things, an article he wrote in the *Medizinische Standeszeitung* (Medical Professional Journal) at the beginning of his medical career, in which he denounced the “Provisionswirtschaft” (payment of commission for referring patients for surgery) and criminal abortion, which had already earned him the enmity of his colleagues at the time ([53]; Fig. 3).^31^

## Librarian of the College of Physicians in Vienna

Following his dispute with Neuburger, Fischer shifted the focus of his work to the College of Physicians in Vienna. In 1921, he became a member of the board of the Society and became its “first librarian”. In this role, he was responsible not only for the library, the most important library of medical literature in Vienna at the time, but also for the Society’s archives [8].

The library contained over 22,000 monographs and 1300 journal titles.^32^ Thanks to Fischer, the Society’s archives were reorganized in the 1920s [8, 32, 54]. At that time, it constituted not only an organizational archive but also possessed an important collection of estates, manuscripts, literature, archival documents, and pictures.^33^ The collection of the Institute for the History of Medicine, which was built up by Neuburger, was fed to a considerable extent by the archives of the Society [15], which at the time had not yet handed over its entire archive despite these donations to Neuburger’s collections, and together with the latter held the most important holdings in the history of medicine at the time.

With its hundredth anniversary approaching in 1937, the College commissioned Fischer to write a history of the Society over this century. Fischer researched the materials available in the Society’s archives and produced a comprehensive manuscript. He was able to present excerpts from this manuscript on the occasion of the Society’s centenary in May 1937 as part of a celebratory lecture in the packed Auditorium Maximum at the University of Vienna in front of an audience of around 500 people, including prominent figures from science and politics such as Austrian Federal Chancellor Kurt Schuschnigg (1897–1977) and the prominent German surgeon Ferdinand Sauerbruch (1875–1951) [8, 55].

Fischer’s history was published in 1938, but without naming an author, as there were fears of complications due to Fischer’s Jewish descent. In the preface to the book, which was dated in late February 1938, the College’s president Eiselsberg thanked the “honorary first librarian” without naming him [56]. Julius Wagner-Jauregg’s (1857–1940) book review published in the *Wiener Klinische Wochenschrift* in mid-1938 also only named the “former first librarian of the College of Physicians” as the author [57].

**Fig. 4:**
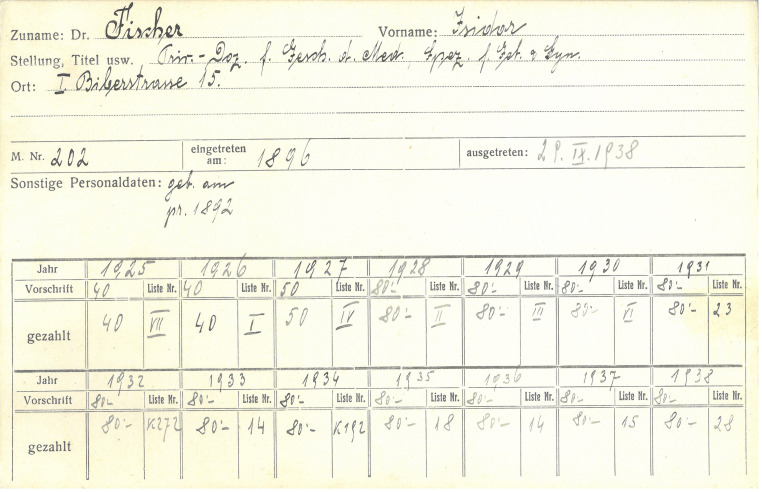
Fischer’s membership card in the College of Physicians. Source: AGDÄ

The practice of no longer naming Jewish authors or publishers on the title page had already been established by the Springer publishing company in Nazi Germany. Although the Viennese branch of the Springer publishing house was not directly affected by Nazi German regulations until the *Anschluss*, it became difficult in 1938 for the Springer publishing company to publish a book with a Jewish author that was also intended for the German market. One factor was that the publishing house owner Ferdinand Springer jun. (1881–1965), as a German citizen, had to comply with the regulations applicable in Germany, even for foreign companies [58]. Fischer managed to flee to London in the fall of 1938, from where he declared his resignation from the Society on 29 September 1938, of which he had been the librarian for many years 34 [8]. His *History of the College of Physicians* remains the reference work on this subject to this day and is cited frequently ([59, 60]; Fig. 4).

## Emigration and death

**Fig. 5:**
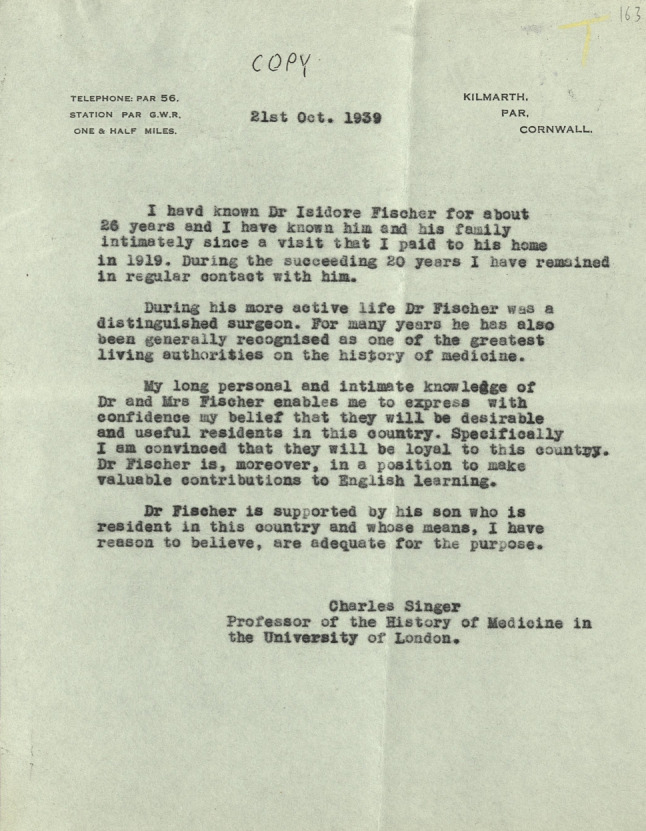
Letter of recommendation for Fischer from Charles Singer. SPSL, MS. S.P.S.L 485/2 1939. Singer to the SPSL, 21 October 1939

Isidor Fischer and his wife Marianne, née Baum (1880–1945), to whom he had been married since 1899 and with whom he had three children, initially found refuge in the UK with their son Leopold Johann, who by then was working as an engineer in London. The family had been torn apart when the Nazis came to power. Leopold had fled the Nazis to Spain in 1935 and was forced to emigrate to the UK from there in 1937. Their elder daughter Josefine Keminka was living in Haifa at the time, where she was working as an obstetrician and gynecologist, like her father. Their younger daughter Alice, who worked as a designer, was living in Paris in 1938 [7]. 35The Fischers’ escape was made possible by Charles Singer and, above all, his wife Dorothea, who later also helped Neuburger escape from Austria at the last minute [2]. Singer generally played an important role in helping refugees fleeing the persecution of the Nazis [61]. Fischer also considered emigrating to his daughter in Haifa, provided that an academic or Jewish organization could grant him a modest monthly stipend until he could stand on his own two feet with his literary activities. Another idea was to emigrate to the USA as a librarian with the support of Oswei Temkin (1902–2002) and Henry E. Sigerist (1891–1957), who, like Singer, had also done much to help Jewish doctors affected by persecution. Fischer approached Temkin with the modest question: “Could the Army Medical Library or the New York Academy of Medicine perhaps use a librarian on a very low salary?”^36^ (Fig. 5).

Singer was able to arrange support for Fischer and his wife from the *Society for the Protection of Science and Learning *and the *Jewish Medical Emergency Association*.^37^ In the UK, Fischer continued his research into medical history and carried out research at the British Museum, the Royal College of Surgeons, and the Royal Society of Medicine [7, 62]. He was also active for the Jewish Medical and Dental Association [2].

In London, the former secretary of the College of Physicians, Dr. Hedwig Pollak, who had also been forced to flee, supported Fischer as his assistant and closest collaborator [49, 59, 62]. She helped Fischer work on his unfinished book *Geographical Names in Medical Terminology* and continued to supervise this work after his death [49]. A typescript of the supplementary volume to the book, which was further edited by Pollak and later by L. T. Norton, is today held in the Wellcome Collection.^38^

In the UK, Fischer also published the important study “British Medicine and the old Viennese School” in the *Bulletin of the History of Medicine*, which brought the history of Viennese medicine to an English-speaking audience [63]. Another publication, “Jews in Modern Medicine” (Medical Leaves, 3 (1940), 30–35), reflects Fischer’s engagement with Jewish medicine, which accompanied him throughout his career. Fischer lived in precarious circumstances. He suffered not only from the consequences of his escape and the lack of financial opportunities, but also from the 6‑month internment of his son Leopold as an “enemy alien” [7]. Sigerist wrote about Fischer in 1940: “Isidor Fischer is, I fear, failing.”^39^ The family eventually moved to Bristol. There, Fischer was diagnosed with Hodgkin’s lymphoma. He died in Bristol on 13 January 1943.^40^ Fischer’s wife Marianne died in London in 1945, where she had returned after her husband’s death.

Following his death, Fischer’s work was received in different ways. While his cultural history approach was embraced in the English-speaking world, his publications on Viennese medicine were particularly well received in Austria. During their exile, the two expelled medical historians, Max Neuburger and Isidor Fischer, made a significant impact. They laid the foundations for a cultural history approach that would become a general trend in English medical history in the 1960s [2]. Research in Austria also built on their work, particularly with respect to the First and Second Vienna Medical Schools [6]; however, a cultural history approach in Neuburger and Fischer’s vein was only implemented to a limited extent in Austria after 1945. In the postwar period, political historiography on the history of medicine in Austria did not go beyond an examination of the period of the Restoration and its aftermath [64], i.e. in principle not really extending beyond the period already examined by Fischer [33, 65].

## Footnotes

1 Archive of the Society for the Protection of Science and Learning (hereafter: SPSL), MS. S.P.S.L 485/2 1939. Singer to the SPSL, Oct. 27, 1939.

2 Josephinum, Ethics, Collections and History of Medicine (hereafter: MUW), MUW-AS-006000-0834. Marlene Jantsch, Manuscript, Overview of the History of Medicine in Vienna. Lecture, WS 1963/64.

3 Sammlung der FB Medizingeschichte, Fischer, Curriculum. Undated (hereafter: Curriculum 1930).

4 SPSL, MS. S.P.S.L 485/2 1939, Curriculum (hereafter: Curriculum 1939).

5 MUW-AS-006014-0035-0007, Fischer to Neuburger, Vienna, April 20, 1924.

6 Curriculum 1939.

7 UAW, MED.PA.588, Personalakt Max Neuburger.

8 MUW-IN-002482, Töply to Adolf Kronfeld, Vienna, October 19, 1932.

9 MUW-AS-006014-0035-0003, Fischer to Neuburger, Partenkirchen, September 19, 1922.

10 MUW-AS-006014-E-0029-024‑1, Eiselsberg to Neuburger, Vienna, September 3, 1917.

11 MUW-IN-002069/123, Neuburger to Sudhoff, Vienna, April 23, 1918.

12 MUW-AS-006014-0035-0003, Fischer to Neuburger, Partenkirchen, September 12, 1922.

13 Ibid.

14 Karl Schmiz (1877–1946).

15 Fischer is mistaken about the location here, as Wilhelm Haberling (1871–1940) obtained his habilitation in Düsseldorf.

16 MUW-AS-006014-0035-0007, Fischer to Neuburger, Vienna, April 20, 1924.

17 MUW-002069/239, Neuburger to Sigerist, Vienna, September 4, 1928, MUW-IN-2069/234, Neuburger to Sigerist, Vienna, November 11, 1928.

18 MUW-IN-002069/26, Neuburger to Sudhoff, Vienna, July 7, 1911.

19 MUW-IN-002069/61, Neuburger to Sudhoff, Vienna, March 31, 1914.

20 MUW-IN-002069/69, Neuburger to Sudhoff, Vienna, July 18, 1914.

21 UAW.Med.Fak.138.ex.1929/30, Zl. 407, Ernennung des I. Fischer zum tit. a. o. Prof. Max Neuburger an den ständigen Ausschuss des Professorenkollegiums der Medizinischen Fakultät Wien.

22 MUW-2069/235, Sudhoff to Neuburger, Leipzig, December 21, 1928.

23 MUW-2069/250, Neuburger to Sigerist, Vienna, December 5, 1929.

24 UAW.Med.Fak.138.ex.1929/30.

25 Curriculum 1930.

26 MUW-AS-006014-0035-0008, Fischer to Neuburger, Vienna, April 23, 1930.

27 MUW-IN-002069/262, Neuburger to Sigerist, Vienna, May 7, 1930.

28 Curriculum 1930.

29 Ibid.

30 UAW.Med.Fak.ex1399/1931/1932, “Artikel im Buch ‘Vienna of Yesterday and Today.’” Fischer to Dean Grassberger, Vienna, August 21, 1932.

31 Curriculum 1930.

32 Archiv der Gesellschaft der Ärzte in Wien (hereafter: AGDÄ), “Bericht über die Bibliothek.”.

33 Ibid.

34 AGDÄ, Akten: 1837-1938, 1938, Fischer to the College of Physicians, Vienna, September 29, 1938.

35 Curriculum 1939.

36 Temkin to Sigerist, Newport, England, August 3, 1938. In: Correspondence Henry E. Sigerist – Owsei Temkin 1931–1956. Edited and annotated by Marcel H. Bickel, Bern 2012, 39-40.

37 SPSL, MS. S.P.S.L 485/2 1939.

38 Wellcome Collection, MS.8062, Geographical Names in Medical Terminology (edited by L. T. Morton and H. Pollak).

39 Singer to Sigerist, Kilmarth, November 3, 1940. In: Marcel Bickel, “Correspondence: Henry E. Sigerist – Charles Singer 1920–1956,” *Medical History* 55 (2011), 205.

40 MUW-HS-001984/11, Obituary Fischer.
